# Translational Advances of Hydrofection by Hydrodynamic Injection

**DOI:** 10.3390/genes9030136

**Published:** 2018-03-01

**Authors:** Luis Sendra, María José Herrero, Salvador F. Aliño

**Affiliations:** 1 Pharmacology Department, Faculty of Medicine, Universidad Valencia, Av. Blasco Ibáñez 15, 46010 Valencia, Spain; luis.sendra@uv.es (L.S.); alino@uv.es (S.F.A.); 2 Pharmacogenetics Unit, Instituto de Investigación Sanitaria La Fe, Av. Fernando Abril Martorell 106, 46026 Valencia, Spain; 3 Clinical Pharmacology Unit, Área Clínica del Medicamento, Hospital La Fe, Av. Fernando Abril Martorell 106, 46026 Valencia, Spain

**Keywords:** hydrodynamic, gene therapy, non-viral, translational

## Abstract

Hydrodynamic gene delivery has proven to be a safe and efficient procedure for gene transfer, able to mediate, in murine model, therapeutic levels of proteins encoded by the transfected gene. In different disease models and targeting distinct organs, it has been demonstrated to revert the pathologic symptoms and signs. The therapeutic potential of hydrofection led different groups to work on the clinical translation of the procedure. In order to prevent the hemodynamic side effects derived from the rapid injection of a large volume, the conditions had to be moderated to make them compatible with its use in mid-size animal models such as rat, hamster and rabbit and large animals as dog, pig and primates. Despite the different approaches performed to adapt the conditions of gene delivery, the results obtained in any of these mid-size and large animals have been poorer than those obtained in murine model. Among these different strategies to reduce the volume employed, the most effective one has been to exclude the vasculature of the target organ and inject the solution directly. This procedure has permitted, by catheterization and surgical procedures in large animals, achieving protein expression levels in tissue close to those achieved in gold standard models. These promising results and the possibility of employing these strategies to transfer gene constructs able to edit genes, such as CRISPR, have renewed the clinical interest of this procedure of gene transfer. In order to translate the hydrodynamic gene delivery to human use, it is demanding the standardization of the procedure conditions and the molecular parameters of evaluation in order to be able to compare the results and establish a homogeneous manner of expressing the data obtained, as ‘classic’ drugs.

## 1. Introduction

The main goal of gene therapy is to use nucleic acids as drugs to treat a wide range of both inherited and acquired diseases. Nucleic acids are large anionic molecules that have great difficulties crossing membrane barriers and usually require the use of vectors or carriers in order to ensure effective transport. Likewise, nucleic acids are easily degraded within biological fluids and this enormously limits their half-life and cell availability. Nowadays, two different types of vectors are being employed to circumvent this situation: viral vectors [1] and non-viral vectors (which are safer but usually less effective).

Since safety is always a priority concern with all drugs destined for human use, it must be underscored that the negative effects associated with these vectors are related to the viral or bacterial sequences that accompany the therapeutic nucleic acids and the carriers (such as proteins or polymers) required to protect them and facilitate their intracellular access.

Although side effects of viral systems are more relevant than in non-viral systems, both types of vectors show toxicity to some degree. In this sense, the hydrodynamic delivery procedure, based on the rapid/pressurized injection of a large volume of gene solution, represents a great breakthrough in the safe management of gene therapy, since it permits the efficient delivery of nucleic acids into the cell without the use of carriers (i.e., avoiding potential toxicity events derived from carriers or vectors). The hydrodynamic procedure has gained renewed interest derived from its potential use in CRISPR/Cas9 gene constructs for gene edition purposes, or in previously formed complexes for transfer to target organs. The procedure would allow access to the cell nucleus, facilitating the interaction of the RNA guide with the complementary sequence and circumscribing CRISPR effects to the target organ alone—thereby partially avoiding the possible systemic effects.

Unfortunately, this successful procedure first developed in murine models is accompanied by important hemodynamic changes in the animal that make it incompatible with clinical practice. This special circumstance makes adaptation of the procedure in large animals a priority for its clinical translation. Overcoming this limitation would be a great advance in allowing the hydrodynamic procedure to be successfully applied to the clinical setting. In the present work, we describe (from our perspective) the efforts made in this field, with the aim of adapting the procedure to achieve therapeutic results, employing suitable experimental conditions for clinical practice. 

## 2. Hydrodynamic Model

During the last decade of the 20th century, different strategies to transfer human genes to mouse hepatocytes in vivo were developed [1], employing both viral and non-viral vectors. Regarding the latter, the viability of the delivery of genes encapsulated in small unilamellar liposomes [1] capable of accessing the hepatocytes was evaluated. The expression of the human alpha-1-antitrypsin (*AAT*) gene was significantly extended when transfection was associated with partial hepatectomy [2] and was improved and prolonged (approximately 50 ng/mL) by employing liposomes encapsulating nuclear location sequences targeted to specific liver cell receptors [3]. However, the final efficacy achieved was several orders of magnitude lower than the values expected to mediate therapeutic benefits. Fortunately, naked DNA transfection employing the hydrodynamic procedure through the mouse tail vein afforded promising results [4]. Later, in 2003, stationary therapeutic plasma levels of protein (>1 mg/mL) during long periods [5] were achieved. The results obtained showed that this new procedure employing naked DNA achieved higher (more than 4 orders of magnitude or 10,000-fold) efficacy than another approach employing non-viral carrier strategies and evidenced that viral and non-viral systems may achieve equivalent therapeutic efficacies. For these reasons, knowing the hydrodynamic procedure and identifying the mechanisms of gene delivery and the corresponding molecular response are of enormous interest in facilitating clinical translation of the method in a precise, safe and efficient manner. In this chapter, the procedure of hydrodynamic injection, its discovery and development and the underlying mechanism of gene delivery are described.

### 2.1. Hydrodynamic Injection of Naked Nucleic Acids

Preliminary studies employing naked DNA evidenced that when using specific conditions for intravascular injection, nucleic acids can be effectively transfected in vivo without any carrier or vector, since:

(a) The DNA reached the muscle and was expressed [6].
(b) The mouse liver could be transfected in vivo by the pressurized injection of a hyperosmotic solution containing naked human growth hormone (hGH) DNA through the portal vein [7], achieving plasma protein levels (65 ng/mL) 50-fold higher than the normal basal values.
(c) High expression levels of tracer genes were achieved in the hind limb muscle of rats by pressurized DNA injection through the iliac artery [8].

**Figure 1:**
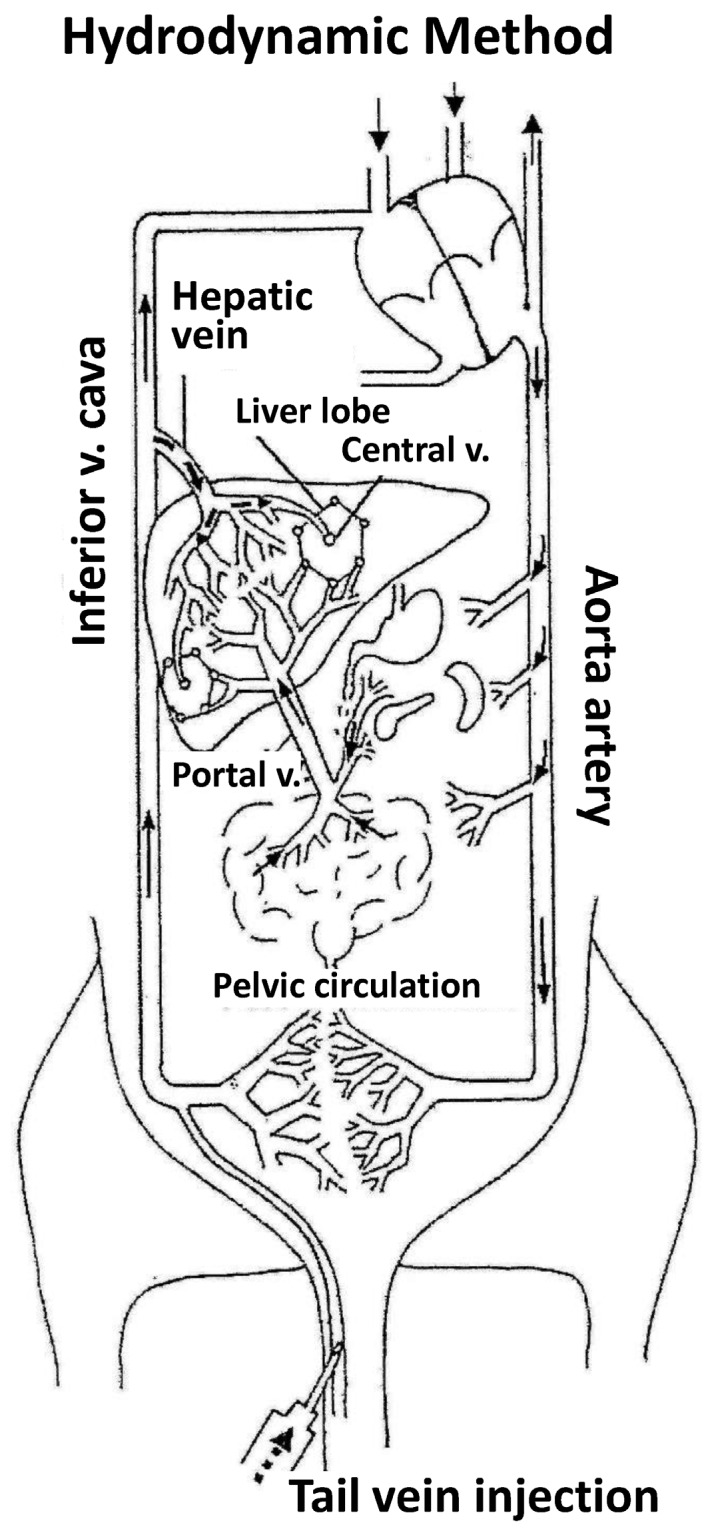
Hydrodynamic method. The figure shows the mouse normal blood flow and the effects mediated by hydrodynamic injection (indicated by a gap line within tail vein and from inferior vena cava to the liver through hepatic vein). Blood flows from tail vein to heart, who drives it to lungs to be oxygenated, returned to heart and distributed to the entire whole through aortic artery. The liver receives a profuse blood supply from the hepatic artery and portal vein. Blood flow from portal vein to inferior cava vein must cross the liver parenchyma through the hepatic sinusoids. When hydrodynamic injection is performed through tail vein, this large volume (2 mL) drains into inferior cava vein, it results in increased venous pressure that mediates retrograde blood flow into liver sinusoids (arrow with dashed line). This permits the gene accessing the liver. Employing different experimental strategies, the hydrofection mechanism has been suggested to involve transient inversion of intrahepatic blood flow and massive fluid endocytic vesicles in hepatocytes, mainly in those distributed around the central vein. The volume stays immobilized but pulsatile until heart, thanks to heart rate increasing, pumps this volume to bloodstream.

The “hydrodynamic procedure” (Figure 1) was definitely described in 1999 [9] as the rapid injection (5–7 s) of a large saline solution volume (1/10 body weight) containing naked DNA through the mouse tail vein. In a typical experiment employing a mouse of 20 g body weight, 2 mL of saline DNA (0.5–5 mg/kg) solution is injected in 5 s. The procedure is especially effective for liver gene transfer [10,11,12] but also for transfer to other organs such as kidney [13], skeletal muscle [14,15] and cardiovascular tissue [16]. After hydrodynamic injection, a rapid increase in plasma levels of liver enzymes AST and ALT is observed, which is quickly normalized within one to three days. On the other hand, the animals tolerate the administration of multiple doses, without apparently affecting liver function or causing any other metabolic effects and/or injuries in other organs [9,14].

The main limitation of the procedure arises from the hemodynamic changes induced by hydrodynamic injection. In the mouse, the rapid injection of 2 mL of saline solution doubles its volemia and consequently results in cardiac pump failure due to the sudden increase in preload.

The hemodynamic impact of injection is important. However, although the animals suffer immediate collapse due to transient cardiac overload, they rapidly recover within the first 5 min. This is due to rapid normalization of cardiac preload, which is possible thanks to the high heart rate of mice. However, these hemodynamic changes would not be compatible with human use and represent the first and main limiting step that must be circumvented in order to allow clinical translation of the hydrodynamic procedure.

### 2.2. Delivery Mechanism

The mechanism underlying the hydrodynamic transfection (hydrofection) of genes in the murine model [10,11,12] has not been elucidated to date. Knowledge of this mechanism will contribute to better understand and adapt the procedure to large animals under conditions compatible with clinical use.

Since the mechanism must be common in all regions, except in relation to some organ particularities (e.g. resilience, vascularization, size, type of vascular endothelium, etc.) that define the injection conditions (volume, flow rate, DNA concentration, etc.) in each case, the present review focuses on the advances achieved in liver gene transfer. It must be underscored that although the experimental stages and results have been clearly confirmed, correct interpretation of the entire process has not yet been corroborated. 

**Figure 2:**
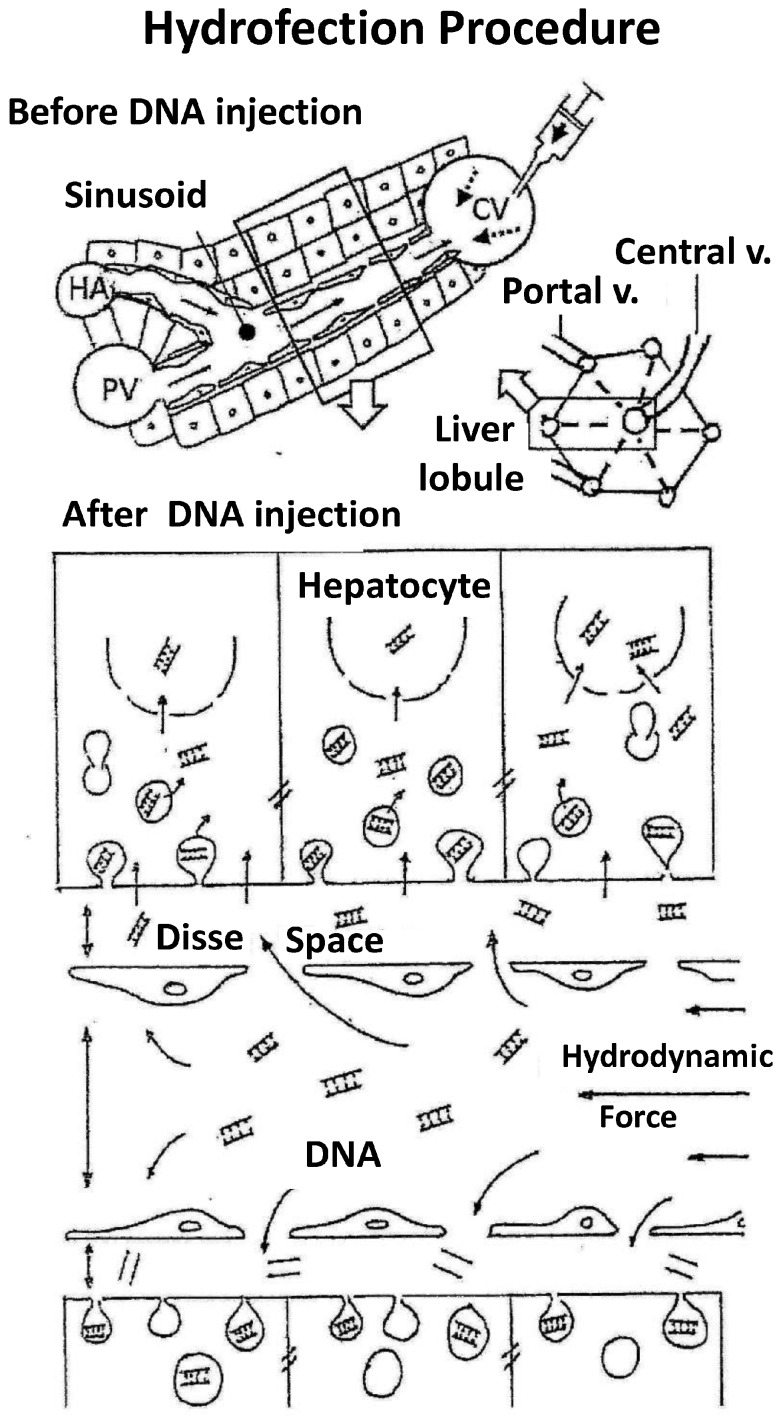
Hydrofection mechanism. The figure shows the mechanism underlying the hydrofection gene transfer to the cells in liver. Upper panel shows a liver lobe unit (right) with its vascular system, which has been enlarged (left) to show the sinusoid vessel detail. Squared area is augmented in lower panel, where the sinusoid vessel organization is showed in detail just after hydrofection. It can be observed that retrovenous injection mediates hydrodynamic force that widens the vessel and virtual Disse space, separates endothelial cells and induces large number of endocytic vesicles on hepatocytes without obvious plasma membrane rupture It suggests that hydrodynamic force mediates DNA delivery to hepatocyte via diffusion process, involving a microfluid uptake process and/or penetration through facilitated permeable sites in the cell membrane. PV: portal vein; CV: Cava vein; HA: hepatic artery.

The main findings to date in relation to the hydrofection mechanism are described below:

(1) The first experiments employing tracer genes and immunohistochemical methods to identify the liver expression of exogenous genes showed expression to be mainly located in central vein areas, thereby supporting that the inversion of flow sense mediates retrograde backflow to the liver and this could be involved in the mechanism of action (Figure 1).
(2) Hydrodynamic injection mediates a remarkable pressure increase in the cava vein, since the tail vein is its direct tributary. This is due to the fact that the administration of a large volume (2 mL in the mouse) in this area (cardiac preload) means doubling the volemia—thereby generating an important pressure increase. This process inverts the pressures at cava vein level with respect to the portal vein and causes retrograde backflow of blood to the liver. This idea is supported by experimental data. The simultaneous measurement of pressures in portal and cava vein areas shows that inversion of portal versus cava pressures exists both during injection and at least 5 min later.
(3) Flow inversion has been observed by intravital microscopy [17] during hydrodynamic injection and minutes later. During rapid injection, flow is inverted and remains static but pulsatile during the first minute. Anterograde flow recovers slowly and progressively until normalization is reached within 5 or 6 min after hydrodynamic injection. It is assumed that the transfection process occurs during this short period of time.
(4) Ultrastructural morphological changes [17] in liver tissue during hydrodynamic injection evidence that the hydrodynamic force exerted upon the liver sinusoids promotes vascular distension, widening sinusoidal pore diameter and the endothelial junctions and facilitating access of the DNA solution to the likewise widened virtual Disse space (Figure 2). This process allows interaction of the aqueous solution with hepatocytes in a high-pressure scenario that promotes the formation of multiple endocytic vesicles without solution of continuity of the cell membrane, as observed by electron microscopy. This excludes the possibility of DNA access to the hepatocyte through membrane disruption and the formation of large permissive pores instead of endocytic vesicles. Moreover, large pores could compromise hepatocyte viability. It must be underscored that the existence of narrow junctions (tight junctions) among hepatocytes limits free DNA diffusion through the intercellular spaces. Thus, hepatocyte hydrofection (gene transfer mediated by hydric forces) could be due to DNA saline solution entry into the hepatocyte, which is mediated by the hydrodynamic force exerted.
(5) Although the possibility that part of the DNA may access the hepatocyte through a receptor-mediated process [7] cannot be discarded, this process would contribute only slightly to the efficiency of hydrodynamic delivery.
(6) The early experimental data, employing molecules of different size and weight, as well as recent observations employing colloidal gold nanoparticles of known diameter and electron microscopy [18,19], support the idea that hydrofection implies a passive process without energy consumption but driven by hydrodynamic force through more permissive sites of the cell membrane, including endocytic vesicles. The dimension of the membrane sinusoids, depending on the species, can be as much as 100 nm in diameter [20]. However, only particles with diameters smaller than 10 nm can access the cell [21], whereas larger particles are virtually refractory to hepatocyte entry but can be observed within the cytoplasm of phagocytic cells (Kupffer cells). This interpretation of the delivery mechanism combines DNA access to the hepatocyte through the cell membrane with no important liver toxicity and justifies the acute plasma increase in transaminases due to marginal cell destruction, which is rapidly reverted within the first days after hydrodynamic injection. This suggests that injured cells are eliminated, whereas the efficiency of transfection remains in those cells in which DNA has gained access in a less aggressive manner. Nevertheless, further studies are needed to establish the exact mechanism of gene delivery mediated by the hydrodynamic procedure.

## 3. Gene Transfer Applications

Gene therapy offers the possibility of treating any type of disease whose aetiology is well defined at molecular level. Once the cause of an illness is known, it is possible to specifically design a gene construct with the ability to improve the pathological scenario and then transfer it with availability for being transcribed and translated. Among the different procedures proposed, hydrodynamic gene transfer in the mouse has been shown to be efficient for transferring genes to different organs in a safe manner and in some cases with high efficacy. This fact makes it possible to expand the potential therapeutic applications of the technique. In this regard, the hydrodynamic procedure for gene transfer has been widely applied for treating a range of diseases in different mouse organs such as muscle and liver. Next, different applications for a wide range of both acquired and inherited diseases are described.

### 3.1. Muscle

The most widely employed procedures for gene transfer to muscle were the direct intramuscular injection and vascular injection of gene constructs through the hind limb vein.

Muscular tissue has served as a target of this therapy since its implantation. Zhang et al. [22] injected a DNA plasmid with the entire murine dystrophin gene into the skeletal muscles of the hind limbs of the Duchenne mouse model. These authors achieved 18–20% of the normal levels of dystrophin protein with 11–16% expressing myofibres. Zhang et al. [23] in turn injected a plasmid encoding siRNA to silence nicotinic acetylcholine receptor α1 to heart and 78% knockdown was achieved 16 weeks after transfection. Bostrom et al. [24] transferred peroxisome proliferator-activated receptor-gamma coactivator-1alpha (*PGC1α*) gene to skeletal muscle and reported protein expression, which improved obesity and glucose metabolism. As a proof of concept, Guess et al. (2013) [25] injected a plasmid encoding tracer siRNA to study the distribution of hind limb gene delivery and observed disperse expression across multiple hind limb muscle groups. Mukumoto et al. [26] compared hydrodynamic liver and intramuscular gene delivery procedures and the mice receiving intramuscular injections yielded better results than mice receiving hydrodynamic injections. Nagata et al. [27] transferred insulin-like growth factor (IGF-1) carried in a polyplex nanomicelle through hydrodynamic hind limb injection in mice with sciatic nerve injury and reported improved motor function for at least 25 days.

### 3.2. Liver

Given the efficiency of hydrodynamic injection in transferring genes to the liver, this was the most widely employed strategy. Different modifications seeking to enhance its efficacy were partial hepatectomy, integrative plasmids and the use of targeting. 

One of the most extensively studied disorders with a potential for treatment with non-viral gene therapy and for which the hydrodynamic procedure has shown the most promising outcomes is probably hAAT deficiency [28]. The entire human *hAAT* gene encapsulated in liposomes of different diameters was transferred to mice. Small liposomes (54 ± 11 nm in diameter) carried the gene to parenchymal cells, mediating the presence of protein in plasma seven days after treatment. Subsequently, Aliño et al. [1,29] reported hAAT protein expression in liver over two weeks, achieving plasma concentrations of up to 160 ng/mL after single-dosing plus liver regeneration induced by partial hepatectomy. Additional studies [2] reported anionic liposomes to mediate more durable protein expression, reaching plasma levels of over 100 ng/mL for more than five months. In 2000, Zhang et al. [4] employed the hydrodynamic procedure [9,30] to transfer the *hAAT* gene and reported the plasma expression of 2–5 µg/mL of protein for six months with peak levels of 0.5 mg/mL. In 2001, Dasi et al. [3] injected *hAAT* gene encapsulated within asialofetuin liver-targeted liposomes, reporting stationary plasma levels of approximately 50 ng/mL of hAAT protein during more than 12 months. The same group [5] transferred the entire genomic *hAAT* gene driven by its natural promoter and reported long-term (>6 months) therapeutic plasma levels (>1 mg/mL) of human protein in mice. Immunohistochemical studies revealed that no more than 5–10% of the liver cells were producing the exogenous protein. Furthermore, it was observed that the injection widened the Disse space and induced the formation of massive endocytic vesicles within the hepatocytes that could facilitate DNA access into the cells. Hydrofection was not only shown to be efficient for *hAAT* gene transfer in mice but also safe [31]—without significantly modifying the levels of liver enzymes or the expression of other genes. 

Haemophilia is another ideal candidate for successful treatment via hydrodynamic gene transfer, since it is an inherited monogenic disease with the liver as specific target organ for gene therapy. Schuttrumpf et al. [32] used human coagulation factor IX in knockout (KO) mice and achieved a plasma expression of 100% normal levels of fully functional FIX protein. In 2009, Keravala et al. [33] transferred phiC31 integrase and the *hFIX* gene to FIX knockout mice. They reported therapeutic plasma levels of protein over six months and functional activity in the correction of bleeding after tail clipping. Likewise, in 2011, Kim et al. [34] reported long-term expression of hFIX (above 500 ng/mL) and clotting activity for seven months in FIX knockout mice after hydrodynamic injection without genome integration. Given the positive outcomes obtained, researchers evaluated gene constructions with greater potential to be employed in humans. In this sense, Schuttrumpf et al. [35] compared the efficacy of transgene expression of classic plasmids versus minicircle vectors in FIX knockout mice over time. Both systems mediated therapeutic plasma levels, with minicircles reaching longer-lasting expression. Having demonstrated the potential application of hydrofection to treat haemophilia B, Matsui et al. [36] employed this strategy to test its possible use in haemophilia A. Accordingly, these authors transferred the entire human FVIII cDNA in a piggyback transposon to mice and reported protein plasma expression (40 mU/mL) for more than 300 days, with functional activity in FVIII knockout mice.

The metabolic disorders diabetes, cholesterolemia and obesity have been the subject of hydrofection studies conducted to evaluate potential applications of the technique to these diseases. In this sense, Holm et al. [37] transferred Short-Chain AcylCoA Dehydrogenase Deficiency (SCAD) cDNA to SCAD knockout mice and reported 30% of SCAD normal levels during 31 days. Another metabolic disease that has been evaluated for treatment using the hydrodynamic procedure is familial hypercholesterolemia. Turunen et al. [38] injected sleeping beauty transposons carrying LDLR and VLDLR to LDLR-deficient mice and observed initial reductions in plasma cholesterol of 17–19%, with significant stabilization of cholesterol levels during at least 6.5 months. Jiang et al. [39] hydrofected leptin and Ciliary Neurotrophic Factor (*CNTF*) genes in mice to evaluate their role in preventing obesity and observed significant reductions in food intake and weight. Mukumoto et al. [26] in turn evaluated the effect of the hydrodynamic and intramuscular injection of a plasmid encoding *IL-6* gene upon diet-induced obese mice. These authors observed lower weight and fat accumulation in treated mice, lesser weight gain and reduced mRNA expression of metabolism-related genes. González-Muniesa et al. [40] hydrofected a plasmid encoding the *UCP-1* gene—a mitochondrial protein with the ability to uncouple mitochondrial respiration—and reported that this strategy can mediate the expression of exogenous genes within the liver mitochondrion, opening a wide range of applications. As an approach to explore the potential application of hydrofection to treat type 1 diabetes, He et al. [41] used insulin cDNA in a plasmid and as a sleeping beauty transposon in diabetic mice and reported the expression of insulin in both plasma and liver tissue, as well as blood glucose reduction and weight loss control. Fukushima et al. [42] transferred the adiponectin gene, which is involved in glucose and lipid metabolism and observed a decrease in circulating glucose (due to the elevated glucose uptake) and triglycerides. Gao et al. [10] also studied the potential use of hydrodynamic gene transfer to treat and/or prevent obesity. Mice fed a high fat diet were treated with *mIL10* gene, whose overexpression prevented weight gain and glucose intolerance. Ma et al. [43] employed a similar model to transfer adiponectin and/or its receptor 2 gene into skeletal muscle and liver and reported similar results—with intramuscular injection yielding slightly better outcomes. Vakili et al. [44] studied the potential benefit of the hydrodynamic procedure for treating type 2 diabetes in diabetic mice. They transferred small hairpin RNA (shRNA) to silence the overexpression of protein tyrosine phosphatase 1B (PTP1B), present in diabetes. The expression of PTP1B decreased by 84% and the plasma glucose levels also diminished. Hydrodynamic injection in experimental diabetic mice has also been used as a screening test to identify therapeutic targets [45]. Gao et al. [11] transferred FGF21 cDNA and evaluated its effect on mice fed a high fat diet—reporting reduced obesity and body weight gain and the alleviation of liver steatosis. In a similar way, Ma et al. [12] injected the *IL-6* gene to high fat diet obese mice in order to evaluate its potential role in the treatment of obesity, observing increased expression of lipolysis genes, a reduction of body weight and improved obesity-associated steatosis.

### 3.3. Other Inherited Diseases

Monogenic inherited diseases are perfect target diseases for gene therapy, since treatment consists of repairing the mutated gene and/or implementing the correct gene to restore the normal phenotype. One of these disorders is mucopolysaccharidosis (MPS), which comprises a group of conditions caused by malfunctioning of lysosomal storage. Camassola et al. [46] injected α-L-idurodinase (IDUA) enzyme cDNA in a model of MPS I mouse and reported efficient transgene expression, which reduced the levels of accumulated glycosaminoglycans (GAGs). In a similar way, Richard et al. [47] transferred the β-glucuronidase gene through hydrodynamic injection or intramuscular injection to treat MPS VII and reported a reduced accumulation of GAGs. In turn, in 2014, Quiviger et al. [48] injected N-sulfoglucosamine sulfohydrolase (SGSH) cDNA in a model of MPS IIIA mouse and observed high serum levels of protein, with the correction of GAG accumulation. Von Willebrand disease (VW) is another inherited disease studied as a target for hydrodynamic gene therapy. Pergolizzi et al. [49] used murine VWF cDNA and achieved the normalization of bleeding time 48 h after gene delivery in mice. Sickle-cell disease (SCD) has also been evaluated as a therapeutic target for hydrodynamic gene transfer. Belcher et al. [50] transferred a sleeping beauty transposon carrying the wild type rat *Hmox-1* gene in SCD mice, which presented hypoxia-induced stasis inhibition. Phenylketonuria (PKU) is a monogenic disease of hepatic origin defined by phenylalanine hydroxylase (PAH) deficiency. Viecelli et al. [51] and Grisch-Chan et al. [52] injected a minicircle encoding PAH cDNA to PKU mice and achieved normalized blood phenylalanine for more than one year. Such long-term expression was suggested to be due to the persistence of minicircles within hepatocytes (probably but not confirmed, as episomes).

### 3.4. Infectious Diseases

The liver can be affected by other acquired and potentially life-threatening disorders. One of the most important is hepatitis, which can have several causes, such as inflammatory alterations, viral infection or a fulminant process characterized by liver inflammation and autoimmune responses. The hydrofection procedure has been employed to create models of hepatitis B (HBV) and hepatitis C (HCV) in mice, making it possible to study the pathology and immune response. In this regard, Yang et al. [53] administered a sleeping beauty transposon carrying the HBV genome and evaluated its expression and the response of the immune system against it. In addition, a model of hepatitis C was developed by McCaffrey et al. [54], injecting HCV genomic RNA. Kim et al. [55] transferred HCV-core-specific siRNA to mice with HCV to limit viral gene expression, silencing viral expression 65–75% two days after injection. Viral infections (hepatitis viruses, adenoviruses) and other causes such as toxins, can produce liver failure. In these cases, the most important concern is to protect hepatocytes from apoptosis. With this purpose, Zender et al. [56] injected siRNAs to silence Cas8, which plays an important role in cell apoptosis and reported one-month survival of mice when treated with anti-apoptosis siRNA versus one day survival in the case of the untreated controls. Fulminant hepatitis and liver inflammation have been studied for gene therapy treatment and researchers have proposed different strategies. In this respect, osteopontin (OPN) has been suggested to increase in fulminant hepatitis and Saito et al. [57] evaluated the benefit of silencing the mentioned gene by injecting anti-OPN siRNA, resulting in amelioration of liver tissue injury. Xu et al. [58] in turn observed that the ectopic expression of B7-H4-Ig fusion protein mediated by hydrodynamic transfer could suppress liver necrosis in mice with induced hepatitis. In a model of hepatitis, Bulau et al. [59] transferred the *IL-37* gene and observed significant reductions in the expression of proinflammatory genes (*IL-1*, *IL-6* and others). Shashidharamurthy et al. [60] showed the hydrodynamic delivery of human FcγR-Ig dimers to block immune-complex mediated inflammation in mice. Anavi et al. [61] employed a model of induced liver inflammation mediated by the impairment of hypoxia-inducible factor 1 (HIF-1). Transfer of *HIF-1* gene to these iNOS knockout mice permitted its expression in liver, ameliorating the liver damage. The NK2GD receptor is another factor related to the initiation and maintenance of liver inflammation. In this regard, Huang et al. [62] employed three specific short hairpin RNAs to silence three major ligands of NKG2D on hepatocytes, achieving the downregulation of those ligands that alleviated fulminant hepatitis. Another strategy applicable in liver inflammation consists of silencing proinflammatory cytokines such as IL-6. In this sense, Gortz [63] injected cDNA encoding a receptor fusion protein to inhibit IL-6 that allowed blocking of the acute phase of liver inflammation. Once the inflammatory process has been stopped and even reverted, liver tissue repair could be necessary. This was explored by Tsai et al. [64], who demonstrated that the overexpression of fibroblast growth factor 7 (*FGF7*) mediated by hydrofection could notably promote liver cell proliferation after partial hepatectomy.

Although the aforementioned diseases have been the conditions most widely studied for treatment through hydrodynamic gene transfer, they are not the only ones. In effect, researchers have evaluated potential application of the procedure to many other different inherited and acquired diseases.

### 3.5. Cancer

One of the major acquired disorders investigated for potential treatment using hydrodynamic gene transfer is cancer. Different approaches have already been adopted. Wu et al. [65] designed a gene construct carrying a member of the TNF family with the capacity to induce apoptosis in specific human tumours. Its transfer in SCID mice with human breast tumours resulted in tumour regression and reduced growth compared to the controls. Yazawa et al. [66] transferred the foetal liver kinase 1 (*Flk-1*) gene, a receptor of vascular endothelial growth factor (VEGF), aiming to block tumour angiogenesis. This permitted a decrease in the formation of new vessels and suppression of the growth of pre-existing tumours. Another strategy involved use of the interferon gamma gene, with the ability to inhibit the formation of metastases. Following this strategy, Miyakawa et al. [67] injected a plasmid encoding a fusion protein formed by IFN-γ and serum albumin and this permitted prolongation of the serum half-life of the protein. Ando et al. [68] confirmed these results and demonstrated that a reduced number of CpG motifs in gene constructs resulted in more durable transgene expression. In 2013, Miyakawa et al. [69] designed another plasmid encoding a fusion protein containing IFN-γ and albumin binding protein that could extend the circulating half-life of IFN, retaining 40–50% of biological activity. With the aim of avoiding the systemic side effects related with IFN, Ando et al. [70] designed constructions encoding fusion proteins of IFN and heparin binding domain in order to target the liver. With this strategy, they achieved tumour growth suppression without systemic adverse effects. Given the interest of immunotherapy strategies against cancer, Ochoa et al. [71] constructed an expression plasmid encoding IL15 and Apo A1 and co-administered it with a plasmid carrying the sushi domain of IL15Rα. These authors achieved an increase in NK cell count, as also reported by Barao et al. [72] and an increase in the number of memory CD8 lymphocytes in blood, spleen and liver—with modest therapeutic effects against colon cancer, for 60 days. Sun et al. [73] evaluated the antitumor activity of IL15/sIL15Rα upon Lewis lung tumour growth in lungs, liver and kidney. They transferred this gene as DNA plasmid, reporting inhibition of tumour growth in all three organs and prolonged survival time. Qiu et al. [74] in turn injected murine IL28B to the liver, aiming to exert its immunomodulatory effect in lungs. This strategy resulted in a decreased expression of inflammatory proteins in mice exposed to cigarette smoke for 21 days.

### 3.6. Other Acquired Diseases

Many other disorders are potentially amenable to treatment with hydrodynamic gene therapy. For instance, Sondergaard et al. [75] employed a hypophysectomised mouse model to transfer the human growth hormone gene. This strategy mediated the normalization of tibia and tail length and body weight gain at the end of the experiment. Lee et al. [76] demonstrated long-term and robust parathyroid hormone expression after transferring a PTH cDNA plasmid containing the OC31 integrase, with transgene integration within the genome. Okumura et al. [77] proved the efficacy of the chemotaxin 2 (*LECT2*) gene in treating osteoarthritis in knockout mice with induced osteoarthritis. Dermatitis has also been evaluated for the application of hydrodynamic gene transfer. Watcharanurak et al. [78] transferred the murine *IFN-γ* gene to mice with early dermatitis and achieved the expression of Treg immunomodulatory cytokines, with improvement of the clinical symptoms. Different types of infection have also been treated with hydrodynamic gene transfer. Wesche-Soldato et al. [79] employed septic mice to transfer siRNA against Fas or caspase-8 and recorded decreased mRNA expression and significantly improved survival. Similarly, Tompkins et al. [80] designed genic constructs to block highly conserved regions of the influenza A virus and inhibit its replication. The treatment of bacterial infections by gene therapy has also been evaluated. Lu et al. [81] reported reduced local skin bacterial counts after human kallistatin transfection in mice previously infected with group A *Streptococcus*.

Given the promising results of the hydrodynamic procedure, other organs and tissues such as the kidneys [82] were studied in Fabry mice. The applications of hydrodynamic gene therapy in small animals are summarized in Table 1.

## 4. Translation of the Hydrodynamic Method: From Mouse to Large Animals

In addition to the murine model, naked gene transfer was employed in rats prior to description of the hydrodynamic procedure. Budker et al. [8] showed that high-pressure injection of naked DNA into the hind limb with occluded outflow mediated high expression levels of luciferase in muscle. In the present chapter, we describe the translational process of hydrodynamic procedure through different animal models.

### 4.1. Rodents

Given the promising therapeutic outcomes obtained with the hydrodynamic procedure, research focused on carrying out the translational process of the technique to large animals, with the aim of achieving clinical application. It must be remembered that mouse hydrodynamic injection involved doubling the volemia of the animal within only a few seconds, which induces cardiac overload that cannot be reverted by large animals—with the result of damaging effects. Most of the studies carried out in rats involved conditions similar to those used for mice. However, different adaptations designed to lower the volume of gene solution have been proposed in order to transfer the translation process to the clinical setting. For this purpose, the original hydrodynamic gene transfer procedure had to be adapted to allow safe application in larger animals. One such adaptation was to target reduced areas of the liver.

In a glomerulonephritis (Th1 predominant disease) model in rats, Higuchi et al. [108] employed a procedure for gene transfer identical to that described for mouse hydrodynamic injection in order to transfer a plasmid containing viral interleukin 10 (pCAGGS-vIL10). These authors observed reduced mRNA expression of disease related genes for seven days. The application of hydrofection was also tested in liver transplantation [109]. For this purpose, CTLA4Ig cDNA was injected in vein, achieving high protein expression that allowed lengthening of graft survival time. Another approach involving the hydrodynamic procedure applied to organ xenotransplantation was described by Miki et al. [110] and consisted of depleting the αGal protein from erythrocytes and kidney by injecting Igκ-EndoGalC (a protein with the capacity to remove αGal). The protein was expressed, with elimination of αGal from the vascular endothelium and digestion of 97% of the protein from erythrocytes for seven days. These effects made it possible to protect the treated rats against specific αGal antibodies. The potential applications of the hydrodynamic procedure are so wide that even neurological disorders have been tested [111]. In this sense, human EPO cDNA was injected through the tail vein of rats and remarkable plasma levels of protein were detected for at least 14 days. Improvement in cell protection against hypoxia injury and apoptosis in neurons was reported. The technique has also been applied in nephrology in order to evaluate its potential use in limiting the fibrotic process [112] triggered after unilateral ureteral obstruction. Antisense oligonucleotides have been injected with the aim of silencing the connective tissue growth factor (*CTGF*) gene. They markedly attenuated the induction of CTGF, fibronectin, fibronectin ED-A and alpha1(I) collagen genes for 14 days, reducing the fibrotic areas. 

Other researchers [113] have explored different strategies to improve hydrofection efficiency by targeting the right lateral liver lobe of the rat through a portal vein branch, co-administering chloroquine to promote endocytic escape and including the gene into nanoparticles approximately 100 nm in diameter. The authors observed transgene expression, such as luciferase activity. Different studies agree that outflow blockade in the target area is needed, since the portal vein pressure is too low to prevent backflow. 

The size limitations of nanoparticles in crossing the cell membrane for gene delivery were confirmed by transmission electron microscopy. The potential application of the hydrodynamic strategy for autoimmune myocarditis was evaluated [114] employing equivalent conditions of gene solution volume. To this effect, the interleukin 1 receptor antagonist (*IL1RA*) gene was injected into the tail vein and 17 days later different clinical parameters such as myocarditis areas, heart weight and heart function were seen to have improved compared with the controls. The expression of inflammation-related genes was also reduced. Employing a similar model of experimental autoimmune myocarditis (EAM) in rats, Chang et al. [115] found proinflammatory IL-17 cytokine to be overexpressed. They injected the immunosuppressant *IL10-Ig* gene and demonstrated that the expression of this transgene suppressed the expression of IL-17 and other proinflammatory cytokines such as TNF alpha and IL-1. Based on the same model, Chang et al. [116] transferred interleukin 18-binding protein (IL18BP). Gene therapy proved effective in controlling EAM, as monitored by a decreased ratio of heart weight to body weight, reduced myocarditis areas, reduced expression of atrial natriuretic peptide, brain natriuretic peptide, IL-17, IFN-γ, IL-6 and IL-10. These effects were likewise suggested to be due to the suppression of IL-17. 

On the other hand, the efficacy of gene expression after hydrofection was evaluated in a model of liver transplantation in rats [117]. Different volumes of gene solution, types of promoters and different doses of luciferase and hAAT plasmids were used. Transgenes were efficiently expressed, with hAAT protein being present in plasma for at least 21 days (with peak levels on day 7); CMV mediated more efficient gene expression. Larger volumes caused more damage, without associated important improvement in gene expression and a limit in gene expression was observed that could not be overcome by increasing the dose of plasmid administered. As already described in application to mice, liver fibrosis is a target disease for hydrodynamic gene therapy and has also been studied in a rat model [118]. Platelet-derived growth factor receptor β subunit (PDGFR-b) was downregulated by gene silencing with siRNA and fibrotic pathways were suppressed, improving the clinical status of the liver. 

As part of the hydrodynamic procedure translational process, automation and reproducibility of gene injection are required. With this aim in mind, Suda et al. [96] designed a computerized injector that was shown to mediate efficient expression of luciferase in mouse (liver), rat (kidney and muscle) and pig (kidney and liver). Having demonstrated the possibility of gene delivery and transgene expression, researchers sought to improve the efficiency of the procedure. In this regard, ultrasound exposure has been found to enhance (up to 4.5-fold) the efficiency of hydrodynamic-based gene delivery for both luciferase and EPO expression within the rat kidney, without altering the histological structure or impairing physiological function of the treated kidney [119]. In another attempt to improve the efficiency of the procedure, the left liver lobe was targeted in rats and outflow occlusion was performed to compare its effect with free-flow control rats [120]. Outflow blockade was described as absolutely necessary to obtain efficient outcomes in transgene expression in several orders of magnitude, as determined by luminescence. Cim et al. [121] studied the potential interest of the procedure in treating type 1 diabetes in rats. The authors aimed to transdifferentiate hepatocytes into pancreatic β cells to produce insulin. The *Pdx1*, *Ngn3* (*Neurog3*) and *MafA* genes were injected, singly and in combination, to livers of normoglycemic rats. Different expression plasmids bearing DNA and mRNA of these genes were used and insulin was detectable in liver for 28 days. Insulin mRNA levels were close to those observed in the pancreas of normal rats 7 days after treatment but declined thereafter. 

It has also been reported that the hydrodynamic procedure may be interesting in application to hypertensive rats. The injection of human hepatocyte growth factor (HGF) has been shown to normalize renal NF-κB activity, proinflammatory cytokines, antioxidant status (GSH, SOD and CAT) and Na+-ATPase activity, reduce renal injury and ameliorate hypertension after 6 weeks of weekly gene administration. The applicability of hydrofection is truly wide and not only circumscribed to the liver, kidney, heart and muscle. Its use in the treatment of peritoneal endometriosis [122] has been evaluated in rats by intravenous injection of a delivery system composed of lipid-grafted chitosan micelles (CSO-SA) and the pigment epithelium derived factor (PEDF). The procedure significantly reduced the size of the endometrial lesions, atrophy and degeneration of ectopic endometrium, without toxic effects. In a model of liver failure in rats, researchers [123] injected *c-met* cDNA and observed transgene overexpression within the hepatocytes accompanied by proliferation enhancement, reduced apoptosis, as well as significant improvement in overall survival. In another study in rat kidney, efficiencies of adenoviral and baculoviral vectors were compared with the efficiency of naked DNA injection. Widespread fluorescent protein expression was observed for more than one month after transgene introduction. Plasmid and adenoviral vectors yielded gene transfer efficiencies ranging from 50–90%, compared with 10–50% mediated by baculovirus vector. 

Liver hydrodynamic gene transfer is able to exert its therapeutic action both in liver and anywhere else in the organism. In this sense, the technique has been found to play an interesting role in the treatment of osteoporosis. The transfer of insulin-like growth factor 1 (IGF-1) gene in an ovariectomized rat model of osteoporosis showed remarkable expression of fluorescence, serum presence of IGF-1 and significant alleviation of osteoporosis. As an interesting approach to the treatment of mitochondrial diseases, Yasuzaki et al. [124] demonstrated that the hydrodynamic gene transfer of naked DNA could mediate gene access to the skeletal muscle mitochondrion after hind limb injection, opening an important field of research.

### 4.2. Rabbit to Pig

The hydrodynamic procedure has achieved promising results in rodents. However, in order to allow clinical application, the translational process and its safety and efficiency had to be demonstrated in larger animals. The problem was that larger animals could not tolerate the demanding hemodynamic conditions of the injection. For this reason, different approaches designed to reduce the volume of gene solution have been proposed, such as targeting a specific organ or a segment of an organ by partially or completely excluding its vascularization. 

In this regard, Eastman et al. [125] injected a reporter gene in rabbit liver using two strategies: (a) targeting a single liver lobe employing a balloon catheter; and (b) targeting the entire organ with hepatic venous occlusion. The authors achieved protein plasma expression over two days, with higher levels when the whole liver was targeted. The safety of liver hydrodynamic gene transfer was also assessed in dogs in order to assess its application to large animals [126]. Four successive injections of 250 mL of hAAT, FIX or Luciferase gene solution were made in four different main liver lobes and numerous variables were evaluated, such as transaminases and cytokines. The authors observed no significant harmful effects and recovery of the animals was rapid.

The following step in clinical translation of the procedure was to test its potential use in animals closer in size and anatomy to humans, such as pigs and primates.

Yoshino et al. [127] and Aliño et al. [128] independently described the first attempts in pigs. They reduced the total volume employed by targeting an area of liver and compared different delivery strategies mediated by catheterization. The strategies tested were: portal vein occlusion, left hepatic artery occlusion, portal vein and left hepatic artery occlusion and the occlusion of both vessels with blood flow washout. Yoshino et al. injected the gene solution (150 mL) carrying *eGFP* or *CTLA4-Ig* gene at 5 mL/s through the cava vein. Occlusion of the portal vein and hepatic artery with washout mediated the most efficient outcomes, achieving disperse fluorescence in liver tissue (due to *eGFP* gene) and CTLA4-Ig protein plasma levels for several weeks, with expression peaks of up to 161 ng/mL one day after treatment. This was the first time the procedure was described in pigs, yielding results of interest for proteins with low levels of expression. In another work, large and small areas of liver were targeted for retrovenous hydrofection in pigs. Aliño et al. [128] reported the presence of gene in liver tissue, as determined by semi-quantitative PCR. Protein expression was also observed by immunohistochemical evaluation in tissue, mainly within the perivenous area. This work also confirmed (by transmission electron microscopy) the previously described [17] formation of endocytic vesicles after hydrodynamic gene delivery in liver. On targeting smaller liver areas while injecting the same volumes of gene solution, the plasma protein levels two weeks after injection reached up to 200 ng/mL—such levels being much lower than those regarded as therapeutic. Fabre et al. [129] targeted the entire liver and isolated the hepatic segment of the inferior vena cava by clamping it supra- and infrahepatically. Gene solution (400 mL) was transferred at 100 mL/s through two parallel syringes and although the efficiency of gene delivery (measured by luciferase) was much lower than that observed in mouse and rat, the authors confirmed the clinical feasibility and safety of the technique as determined by systemic blood pressures, electrocardiography, heart rate, etc.

In parallel to the development of liver hydrofection in large animals, other possible applications of the procedure were also tested. In this regard, cardiac gene transfer by an adaptation of the hydrodynamic procedure was studied [18]. A naked *eGFP*-carrying plasmid was injected (50 mL; 20 μg/mL) through the coronary sinus at 5 mL/s employing a catheter, while another catheter fitted with a balloon was placed proximally within the sinus to block outflow. This procedure yielded efficient gene delivery (1–200 copies of gene per haploid genome), exhibiting a relative transcription rate with respect to the *GAPDH* gene of 0.2–10 mRNA copies. The expression of protein was observed by immunohistochemistry. That same year, the efficiency of the hydrodynamic procedure in application to skeletal muscle gene transfer in pigs [130] was demonstrated by the expression of luciferase after gene transfer (100 μg/mL) in the hind limb. In the hands of these authors, the injection of 300 mL (1.5% of body weight) of luciferase gene solution at 15 mL/s and 300 psi pressure yielded 10^6^–10^7^ RLU/mg of protein 5 days after injection and gene expression in transfected cells was maintained for two months. Given the important role of the pressure reached within the liver during hydrodynamic injection, Fabre et al. [131] focused on pressurizing individual liver lobes by excluding their vasculature. Seeking to achieve localized high-pressure levels without affecting the systemic circulation, these authors proposed individualizing the lobes by employing catheters with balloons and ligation. Luciferase gene (200 mL) was injected through individual branches of the portal vein to targeted liver lobes at 50 mL/s, with occlusion of their tributary suprahepatic cava vein and without occluding normal portal flow and cardiac load. This strategy allowed the reaching of intrahepatic pressure levels of up to 100 mmHg. 

**Figure 3:**
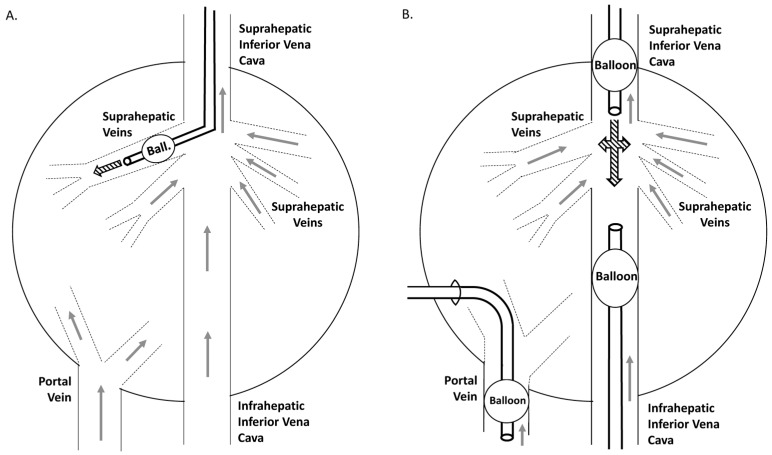
Catheterization strategies for minimally invasive liver hydrofection ‘in vivo.’ Schematic representation of liver venous vasculature and catheters position. Continuous line represents extrahepatic vessels. Gapped lines represent intrahepatic vasculature. Grey arrows indicate the normal blood flow sense. Black thick arrows indicate the sense of hydrodynamic injection.

Although most authors have pointed to blood pressure as the most important feature of hydrodynamic injection in mediating efficient gene transfer, other investigators have described other characteristics of injection, such as impulse [84] and flow rate [132,133], as being of relevance. In any case, almost all authors agree on the need or benefit of isolating target areas or the entire liver by vascular exclusion in order to improve the efficiency of the procedure. Such vascular isolation may be partial or complete. It had been previously reported that the complete liver vascularization of the pig could be occluded for up to 20 min without hepatic injury or systemic damage [134]. Based on this fact, Carreño et al. [132] described a surgical procedure for completely sealing the pig liver in vivo and performing the hydrofection targeted to the whole organ. An *eGFP* gene (20 μg/mL) saline solution (200 mL) was injected at 10, 20 and 60 mL/s simultaneously through the suprahepatic inferior cava vein and the portal vein using two catheters connected by a Y-connector and a high-volume pump. Optimal conditions were found to be the retrograde injection of 200 mL at 20 mL/s. Due to the invasiveness of the surgical procedure, same authors adapted this model for sealing the liver venous system through catheterization [21]. Two strategies (Figure 3) were proposed and compared: (a) the use of a balloon catheter for insertion into a single lobe; and (b) the use of three catheters with balloons simultaneously placed within the supra- and infrahepatic inferior cava vein and in the portal vein, around entry to the liver. The procedure involving complete venous obstruction and entire organ targeting adopting the suprahepatic inferior cava vein route yielded greater gene delivery, transcription and translation in liver tissue 14 days after injection—achieving more than 10^5^ copies of hAAT protein per cell. However, the presence of protein in plasma was dramatically lower (more than 4 orders of magnitude)—a fact that could be due to differences between species. Catheterization-mediated hydrodynamic gene delivery in a single lobe was also used to compare the efficacies of the naked DNA and foamy viral vector strategies [135]. The *eGFP* gene was transferred in a naked plasmid and in a foamy viral vector. The presence and expression of *eGFP* was determined one week and one month after transfer by PCR and qPCR versus *GAPDH*, assessed as ΔΔCt. The authors recorded better outcomes using viral vector, with expression levels of up to 29.7% of the endogenous *GAPDH* levels.

Open procedure model (left panel): catheterization was carried out through the jugular vein with an 8 Fr balloon catheter placed in a suprahepatic branch. 

Closed procedure model: The catheter-mediated liver vascular isolation was performed by simultaneous catheterization of the intrahepatic portal vein (transhepatic catheterization with a 10 Fr balloon catheter) and both the supra- and infrahepatic inferior cava vein (two 8 Fr balloon catheters through the jugular and femoral veins), with balloons limiting the perfusion area. This permitted excluding the entire liver venous vasculature.

### 4.3. Primates

Different gene therapy attempts have been carried out in primates [93], with the aim of transferring genes to skeletal muscle, through the hind limb with occluded outflow. This strategy proved efficient for the expression of plasmid DNA and/or siRNA, as determined by luciferase quantification. Wooddell et al. [98] tested the expression efficacy of different LacZ tracer gene constructs driven by different promoters in mice, rats and rhesus monkeys. They determined the long-term (up to 49 weeks) expression of LacZ in mice, with better outcomes being recorded when employing the CMV promoter. Despite the different gene therapy studies carried out in primates, most of them [136,137] have employed viral vectors to transfer genes to skeletal muscle and liver. However, the ethical implications, the lack of clinical response (possibly limited by viral particle size) and the difficulty of working with this animal model have limited its use and researchers have focused especially on pigs. 

The applications of hydrodynamic gene therapy in middle to large size animals are summarized in Table 2 and Table 3.

## 5. Parameters of the Genes Transfer Process

The efficacy of gene transfer can be measured by different procedures and authors have studied many variables to present their results and evaluate how efficient a procedure is and how interesting it could be for clinical application. When the murine model and standard efficient hydrodynamic injection is employed, protein translation is efficient and quantifiable both within tissue (immunohistochemistry, Western blot, ELISA) and eventually also in the bloodstream (ELISA). After confirmation of the optimum response of hydrodynamic gene therapy in the murine model, the development of new animal models, different delivery strategies and gene constructs, etc. involving less efficacious protein production, required other parameters for the expression of results. In this regard, a detailed analysis was needed to establish the effectiveness of each stage of the gene decoding process. Such evaluation was able to identify the steps limiting the efficacy of the procedure. In this chapter, we suggest a quantitative manner of evaluating the molecular process of gene decoding that could facilitate the comprehension and reporting of gene expression results. 

The molecular evaluation of decoding is mandatory for correct interpretation of the process. The determination of DNA, mRNA and protein can be qualitative, semi-quantitative or quantitative. Qualitative analyses only allow identification of the presence of a molecular species, without possible interpretation of the efficiency of the procedure, gene construct, etc. The semi-quantitative determination of nucleic acids has been widely employed and consists of expressing the relative amount of DNA or mRNA based on the presence of a housekeeping gene. This strategy has limitations that impede correct evaluation and comparison of different procedures, such as differences in the housekeeping genes employed or different gene expressions depending on the cell type and situation involved—thereby precluding the obtainment of real information on the amount of the specific molecular species. Western blot is used for the semi-quantitative determination of protein. This technique allows us to observe the presence and approximate amount of protein by comparison with other samples or purified protein in known quantities. Quantitative determination of the molecular process in turn affords real data on delivery, transcription and translation indexes expressed as the number of copies per cell of the respective molecular species. In the case of DNA and mRNA, real-time quantitative PCR is required. The data obtained for samples must be plotted on a standard curve prepared with a defined amount of the same gene construct injected. For protein quantitation, the ELISA technique with a standard curve prepared with serial dilutions of the purified protein is performed. By knowing the molecular weight of each molecular species, the exact number of molecules present in a unit volume and/or weight can be determined. Different reference units have been proposed: total protein weight, total organ weight, total DNA/RNA/protein, etc. In order to facilitate comparison of the results and objectively define the best conditions for gene transfer, authors should reach consensus on data quantitation and expression. 

When a secretion protein is employed and its presence in plasma is evaluated, we suggest that the pharmacological term efficacy should be maintained, defined as the amount of protein in plasma per dose of DNA employed. The results should be expressed in molecular units (e.g. number of copies or moles) whenever possible or opportune, or alternatively in units of mass. This would make it possible to establish the optimum doses for potential treatment. We suggest evaluating the expression of transfected gene in a quantitative and objective manner, employing a universal standard reference. Since the cell is the biological reference unit, we consider that the amount of DNA, RNA and protein should be related to a normalized cell. When the results are evaluated in cultured cells or tissues, the data should be referred to a common circumstance such as a standard or “normalized cell.” Such data make wide sense in the majority of studies from different areas. A normalized cell has been described [142] as having the following average characteristics: weight 3–4 ng with a volume of ≈1 pl and a diameter of ≈10 μm, depending on the cell type. The total protein content of a cell is ≈700 pg but the dynamic range of concentration can span up to 7 orders of magnitude [143]. The mass of genomic diploid DNA in a human cell is estimated to be ≈6.6 pg, whereas that of RNA is ≈10–20 pg [144]. 

A normalized diploid cell was classically described by Alberts et al. as a typical mammalian hepatocyte with a defined content of total DNA (genome weight of the specific animal, human: 6.6 pg), RNA (20 pg) and protein (500 pg)—and these are the parameters we employed as reference. 

Three types of parameters can be used in seeking to objectively assess the efficiency of the gene transfer procedure step-by-step, minutely at molecular level:

(a) Indexes, for the absolute number of copies of each molecular species (DNA, RNA or protein) referred to a normalized cell.
(b) Intrinsic activities, representing the index ratio between consecutive steps (transcription (RNA/DNA) and translation (protein/RNA)) of the decoding process, in order to evaluate how efficient each step is, in a normalized cell.
(c) Expression efficacy, defining the final efficacy of the procedure in the tissue relating the amount of protein copies per gene copy, in a normalized cell.

The formulas of each parameter can be defined as follows:

Index formulas: 

**-** Delivery index: transgene DNA copy number/animal diploid genome weight. Example for humans: 

Delivery index = X transgene copies/6.6 pg of total DNA

**-** Transcription index: transgene RNA copy number/20 pg of total RNA. Example for mammalian cells:

Transcription index = X transgene mRNA copies/20 pg total RNA

**-** Translation index: transgene protein copy number/500 pg of total protein. Example for mammalian cells:

Translation index = X transgene protein copies/500 pg total protein

Intrinsic activity formulas: 

**-** Transcription activity: (transgene RNA copy number/20 pg of total RNA)/(transgene DNA copy number/animal diploid genome weight). Example for human cells:

Transcription activity = (X transgene mRNA copy number/20 pg of total RNA)/(X transgene DNA copy number/6.6 pg total DNA)

**-** Translation activity: (transgene protein copy number/500 pg of total protein)/(transgene RNA copy number/20 pg of total RNA). Example for mammalian cells:

Translation activity = (X transgene protein copy number/500 pg of total protein)/(transgene RNA copy number/20 pg of total RNA)

The efficacy of gene expression relates the protein translation index to the gene delivery index in order to define the global efficacy of the procedure. 

The formulas referred to gene expression efficacy are defined as: 

- Expression efficacy: evaluated from organ tissue or cell culture.

Expression efficacy = (transgene protein copy number/500 pg of total protein)/(transgene DNA copy number/animal diploid genome weight)

- Efficacy: evaluated from plasma or extracellular fluid. Classically, final efficacy is expressed as plasma concentration of protein (w/v) related to the dose of DNA (w) administered (protein (w/v)/DNA (w)). In order to easily relate this with the abovementioned parameters, the dose and concentration should be expressed in moles (protein (mol/v)/DNA (mol)). 

Efficacy = concentration of protein (w/v)/dose of DNA (w) administered.

We consider this strategy to offer an objective and clear analysis that enables expression of the data as a ratio of copy number of each molecular species (regarding the usual content of DNA, RNA and protein) in a normalized cell. This offers a more comprehensive and visual interpretation of the entire process and allows comparison of the results from different works and research groups. Furthermore, the intrinsic activities and expression efficacy allow us to identify the limiting steps of the decoding process and establish optimum doses for treatment. 

## 6. Hydrodynamic Genes Transfer to Human Organ Ex Vivo

The hydrodynamic procedure has been shown to be highly efficient in the murine model, achieving therapeutic plasma levels of human proteins of clinical interest. Clinical translation to larger animals has been partially disappointing in terms of final protein secretion. This lack of efficiency has been reported to be due to interspecies differences during protein folding and/or exportation. However, positive results in tissue gene decoding have been recorded, showing effective protein translation within liver tissue. The translational procedure in animal models has been completed and this limitation could only be circumvented by employing human tissue. Thus, application of the hydrofection strategy in human ex vivo models is justified, since this could complete the translational process prior to implementing a clinical trial. In the present chapter, different adaptations for employing human tissues as a target and solve the obvious limitations of in vitro models are described.

### 6.1. Isolated Human Organ Segments

Liver segments from surgical resection are a good option, since they have the required features for optimum hydrofection: the vasculature is preserved and catheters can be placed within them. Furthermore, retrograde injection through a suprahepatic vein can be performed and complete sealing is established due to the surgical intervention. Herrero et al. [145] employed such human liver segments to retrogradely inject a eGFP plasmid (20 μg/mL) driven by a CMV promoter through a short 9 Fr catheter placed in a suprahepatic vein. Different injection conditions were compared to establish the most appropriate ones: volumes equivalent to 1/5 or 1/10 segment weight and flow rates of 1, 10 and 20 mL/s. 

Protein expression and tissue distribution, evaluated by fluorescent and immunohistochemistry studies, showed good and wide gene expression and the quantitative molecular analyses reinforce the histological findings. Employing similar human liver segments from surgical resection, Sendra et al. [146] hydrodynamically injected a volume equivalent to 1/5 of the liver segment weight of interleukin-10 plasmid solution (20 μg/mL) at 10 and 20 mL/s. Interleukin-10 protein is not produced naturally in liver and its levels within liver tissue are low. For this reason, the presence of IL10 protein within liver could be considered as produced by the plasmid hydrofection. Regarding the results previously reported by our group [147] in pig heart in vivo, the indexes referred to tissue IL-10 expression (over 100 copies/cell [145,146]) could mediate local immunosuppressant pharmacological effects of interest for controlling the immune response (the IC50 of IL-10 for TNFα being 124 pg/mL [148]) triggered after liver transplantation. 

This model allowed evaluation of the efficiency of human gene transfer and decoding in human liver tissue. Given the interest of this strategy for transferring genes to human organs, the human liver segment hydrofection procedure was adapted [149] to human colon segments. The segments from surgical tumour resection maintain the complete vasculature of the segment, allowing injection of the gene solution under hydrodynamic conditions. The *hIL-10* gene (20 μg/mL) solution was retrogradely injected in tumour-bearing colon segments (50 mL at 20 mL/s) and the presence of IL-10 DNA, RNA and protein was quantified. The procedure mediated a protein translation index in tissue of up to 1000 copies/cell—this being close to the values obtained in liver. As a preliminary experiment, one colon segment with inflammatory bowel disease was also used [149] to evaluate the potential interest of the procedure in treating disease. The procedure mediated similar levels of tissue protein translation (around 1000 molecules per cell). This production of protein could have immunosuppressant pharmacological effects of interest for the treatment of inflammatory disorders. The applications of hydrodynamic gene therapy in humans are summarized in Table 4.

The next step consists of employing this human organ segment model to transfer a human gene of clinical interest for treating disease. With this purpose in mind, we used the previously reported conditions to transfer the human *AAT* gene to human liver and evaluate its decoding efficiency in its target tissue. Aiming to completely differentiate between the protein encoded by the transferred gene and the endogenous AAT protein (with high rates of liver production), special gene constructs bearing an additional tracer (tag/flag) sequence of DNA with the ability to be transcribed and translated into synthetic mRNA and protein were required. In this regard, we have designed and constructed a plasmid containing the entire *hAAT* gene with a flag sequence (hAAT-flag) presenting an open reading frame to permit its transcription and translation. We have already conducted preliminary studies in four human liver segments, injecting this AAT gene plasmid containing the flag sequence (hAAT-flag), under the conditions used in other studies. After gene transfer, tissue samples were cultured at 37° under a 5% CO2 atmosphere in DMEM medium. The analyses of the gene decoding process showed efficient expression of hAAT-flag protein in liver tissue (Table 5). The amount of tagged protein (AAT-flag) accounted for close to 50% of total tissue AAT three days after gene transfer. This result proves that both endogenous and exogenous proteins were expressed with similar efficacy and that consequently the relative expression efficacy of exogenous protein is 100% when compared with the endogenous protein. This demonstrates that the hydrofection procedure could mediate efficacious protein expression after exogenous gene transfer in humans. The efficacy of protein export into the bloodstream from liver in order to exert systemic effects remains to be elucidated and further experiments and models would be required.

### 6.2. Future Perspectives: Isolated Organ with Continuous Vascular Perfusion and CRISPR Gene Edition

Hydrodynamic liver gene therapy offers the possibility of treating systemic disorders due to the capacity of the liver to produce and release proteins into the bloodstream. Thus, a new model allowing the study not only of decoding but also of export should be developed. An isolated organ model with vascular perfusion and the possibility of keeping an entire human liver viable for a few days should allow us to evaluate the entire decoding process after hydrofection, from delivery to protein translation and export into the circulating medium. At present, different devices for maintaining liver circulation ex vivo are being used in the field of liver transplantation, such as Organox^®^ and other experimental systems have been proposed [159]. Our group has already designed and constructed a preliminary system offering the possibility of liver maintenance for up to two days for preclinical studies. The hAAT-flag plasmid could be used in human livers connected to a continuous perfusion machine, with evaluation of the secretion of a human exogenous protein by the human liver. Efficient expression and secretion of hAAT-flag protein in perfusion medium could constitute the last step in the translational process prior to use in a clinical trial.

Hydrodynamic procedure for gene transfer has normally been used for naked DNA transfection. However, this strategy could facilitate the delivery of other gene constructs containing viral and non-viral vectors. Hydrofection would permit overcoming the main limitation of the different strategies for genome editing or repair (Zinc fingers, TALENS). In recent years, a new strategy (CRISPR/Cas9) for genome editing in a customized and inexpensive manner has emerged [129,130] and this technology is reaching many laboratories. Although is an efficient strategy for targeted genome edition, a number of limitations must be resolved—fundamentally the difficulty of delivery to target cells and/or organs. The great appearance of CRISPR/Cas9 and its wide application throughout the world gave a renewed interest to hydrodynamic gene transfer methodology. The hydrodynamic gene transfer could mediate the delivery of CRISPR/Cas9 constructs in vivo, both as plasmid and as ribonucleoprotein complex, as a safe and efficient procedure of transfection.

## Figures and Tables

**Table 1:** Hydrodynamic gene transfer works performed in murine model. The table shows the author of each work, the publication year, the target organ assayed, gene and gene construction, conditions of hydrodynamic injection, experimental aim and methodology, disease of interest and effect duration.

Author	Year	Organ	Gene	Gene Construct	HD Variants	Experimental Aim	Disease	Long-Term Expression
Bell, J.B. [83]	2007	Liver	HemOxigenase-1, *LDLR*/*VLDLR*	SB Transposon	HD	Transgene expression, qRT-PCR, Integration, Plasma presence, Clinic variables	Method, Sickle-cell disease, Familial hypercholesterolemia	up to >6 months
Belcher, JD [50]	2010							
Hackett, P.B. [84]	2011							
Turunen TA [38]	2016							
Yang, PL [53]	2002	Liver	HBV	Sleeping beauty transposon-super genomic DNA	HD-HBV induction	Histology, titration, southern blot, northern blot, PCR, ELISA	Hepatitis B	20 days
Doherty J [85]	2012	Liver/HEK293, HeLa, T cells	Neomycin resistance, full length Factor VIII	PiggyBac Transposon Vector cDNA	HD	Transgene expression, qPCR, clinical assays	Method, Haemophilia A	up to >300 days
Matsui H [36]	2014							
Chen, I.Y. [86]	2014	Liver/heart	*Luc*	Titrable two-step transcriptional amplification vector strategy	HD/intramyocardial injection	Fluc exp modulation by raloxifene-mediated activator		
Camassola M [46]	2005	Liver/syst	*IUDA*	Superfect complexes cDNA	HD caudal vs. intraperitoneal	Activity, transgene expression, PCR	Muccopolysacharidosis I	14 days
Alino SF [1]	1994	Liver	*hAAT* (pTG7101)	Small liposomes	single & multiple dose + Partial HTx	ELISA & Liver Cytophotometry	15 days	
Zender, L [56]	2003	Liver, heart, vascular	*Cas8, alpha-gal, antiviral, Fas, Cas8, GFP, OPN, nAchRalpha1, Apo-LP*	siRNA	Intraportal, aortic, in vitro	Luciferase, expression, Western Blot, ELISA, immunofluorescence, survival, virus titre, histochemistry	Liver failure, Influenza, sepsis, hepatitis, transplantation, atherosclerosis	up to 4 months
Tompkins, SM [80]	2004							
Chu [87]	2005							
Wesche-Soldato, DE [79]	2005							
Saito Y [57]	2007							
Zhang G [23]	2011							
Kim, S.I. [55]	2009							
Wesche-Soldato DE [88]	2008							
Huang M [62]	2013	Liver	*NKG2D, PTP1B*	shRNA	HD	Cell count, histology, cytometry, Luciferase, Western Blot, qPCR, glucose levels	Hepatitis, diabetes	up to 10 days
Vakili S [44]	2013							
Magnusson T. [89]	2011	Liver	*Luc*	Promoter: CMV-EF1a	HD-tail vein	Luciferase, qPCR		up to 2 months
Schuttrumpf J [35]	2011	Liver	FIX human	Plasmid vs. minicircle	HD	Expression, function, methylation	Haemophilia B	100 days
Ando M [70]	2014	Liver, kidney, muscle, lung, cells, tumour	*IFN-heparin binding domain, hAAT, hGF, PTH, alpha-Gal, hFIX, IFN-alb, hIL37, Luc, FcgammaR-Ig, PAP1, mIL10, mKATE, hKS, IL28B, mIL15+mIL15R, hGH, EPO, Distr, LacZ, VWF, Flk-1, UCP, Adiponectin, IGF, IFN-albumin binding peptide*	Plasmid DNA	HD, retro-orbital, hind limb, im, kidney injection, saphenous vein	Serum concentration, expression, ELISA, qPCR, Western Blot, histology, Luciferase, glucose levels, injury, electron microscopy	Cancer, hAAT deficiency, hypophysectomised, hypoparathyroidism, Fabry disease, Haemophilia B, metastasis, hepatitis, pancreatitis, obesity, dystrophy, inflammation, hGH deficiency, streptococcus infection, method, Von Willebrand disease, diabetes, nerve injury	up to >8 months
Zhang G [4]	2000							
Sondergaard, M [75]	2003							
Lee S [76]	2008							
Nakamura G [82]	2008							
Kim H.S. [34]	2011							
Miyakawa N. [67]	2011							
Bulau, AM [59]	2011							
Yan S [90]	2012							
Shashidharamurthy, R [60]	2012							
Shigekawa, M [91]	2012							
Gao M [10]	2013							
Guess, MG [25]	2013							
Lu S.L. [81]	2013							
Qiu C [74]	2014							
Sun H [73]	2016							
Wolff, JA [6]	1990							
Dagnaes-Hansen, F [92]	2002							
Alino, SF [5]	2003							
Hagstrom, JE [93]	2004							
Zhang, G. [94]	2004							
Crespo, A [17]	2005							
Pergolizzi, RG [49]	2006							
Yazawa, H [66]	2006							
Gonzalez-Muniesa, P [40]	2006							
Fukushima, M [42]	2007							
Li, W. [95]	2008							
Schuttrumpf, H [32]	2008							
Suda T [96]	2008							
Podetz-Pedersen, KM [97]	2010							
Xu, JF. [58]	2010							
Herrero, M.J. [31]	2011							
Ma, Y [43]	2013							
Miyakawa, N. [69]	2013							
Wooddell, C [98]	2011							
Nagata, K [27]	2014							
He, C [41]	2004	Liver/Skeletal Muscle	*Insulin, hGF, IL6, IFNg, mFGF21, SGSH, IL6-RFP-Fc, IL6*	Plasmid cDNA	HD, im	Expression, plasma protein, immunohistology, clinical analysis, pathology, qPCR, WB, ELISA	Diabetes type 1 and 2, obesity, glomerulonephritis, dermatitis, MPSIIIA, inflammation	up to 120 days
Bu, X [99]	2011							
Mukumoto, H [26]	2013							
Watcharanurak, K [78]	2013							
Baribault, H. [45]	2014							
Gao, M [11]	2014							
Quiviger, M [48]	2014							
Gortz, D [63]	2015							
Ma, Y [12]	2015							
Duguid, JG [100]	1998	Cell lines	*b-Gal, hGH, eGFP*	Peptide/DNA pH sensitive, PEI & DOTAP/DNA complexes	in vitro	Cytochemistry, g-gal chemoluminiscence, fluorimetry, electrofluorescence, TEM, cytofluorescence, dynamics of gene transfer		14 days
Moret, I [101]	2001							
Alino, SF [102]	2000					PD:D-R, Em, EC50, Pot, Afin		
Alino, SF [28]	1993	Liver	*hAAT*	Large/small liposomes, Liposomes (-)vs(+) plus	iv, HTx	Cytophotometry, DNA, Size Distribution, ELISA		up to 5 months
Alino, SF [5]	1993							
Alino, SF [29]	1996							
Crespo, J [2]	1996							
Budker, V [7]	1996	Liver	*b-Gal, hGH*	Naked	DNA, hypertonic solution-portal injection, hepatic vein occlusion	ELISA & histology		2 days
Xu, Z.X. [103]	2009	Liver	*hFIX*, *hAAT*	Integrative DNA plasmid	HD	Specific insertion, plasma concentration, toxicity, expression, IHC	Haemophilia B	up to 250 days
Keravala, A. [33]	2011							
Ando, M [68]	2012	Liver	*IFN*	pDNA varying CpG motifs number	HD	Expression	Cancer	
Viecelli HM [51]	2014	Liver	*mPAH*	Minicircular cDNA	HD	Expression serum and tissue, qPCR, histology	Phenylketonuria	>1 year
		Liver	HCV, others	Genomic RNA-HCV internal ribosome entry site firefly luciferase, Non-viral	HD-HCV model	Histology, Luciferase	Hepatitis C	10 days
McCaffrey, AP [54]	2002							
Habbitt, OC [104]	2007	Liver	gDNA (100 kb), *eGFP*, *LDLR*	gDNA, GenomicGenes, BAC	HD	Efficacy vs. DNA copy number	Cholestrolemia	4 months
Okumura, A [77]	2008	Liver	*LECT2*	Expression vector non-viral	HD	Inflammatory expression, histopathology, PCR	Arthritis	12 days
Zhang, G [22]	2010	Muscle	full-length Dystrophin Gene	Full length DNA	HD-limb vein	Distribution, expression, myofibres damage, Western Blot	Duchene	
Shahaf, G [105]	2011	Liver	*hAAT*	Epstein Bar Virus-plasmid	HD	Islet function, Treg, macrophage, IL1	Islet allogenic transplant	up to 100 days
Ochoa, M [71]	2012	Liver	*IL15+ApoA1+IL15Ra*	Expression plasmid cDNA	HD	Cell count, pathology, Western Blot, PCR, cytometry	Cancer	60 days
Holm, DA [37]	2003	Liver	*SCAD*, promoter genomic elements	cDNA	HD, in vitro	Plasma protein, NK reconstitution, toxicity	Metabolic disease	31 days
Barao, I [72]	2011						Immunodeficiency and transplantation	18 days
Hibbit, O. [106]	2011							
Dasi, F [3]	2001	Liver, Plasma	*hAAT*	ASF-Lp, PS, DOTAP, NLS	iv + Partial HTx	ELISA, PCR, Sequencing	hAAT deficiency	6 months & 12 months
Wooddell, CI [107]	2008	Liver	Alkaline Phosphatase Reporter gene	Albumin promoter	HD	Plasma protein	Method	Albumin 1 year vs. CMV 1 day

**Table 2:** Hydrodynamic gene transfer works performed in mid-size animals. The table shows the author of each work, the publication year, the animal model studied, the target organ assayed, gene and gene construction, injection methodology, variables evaluated, disease of interest and effect duration.

Author	Year	Species	Organ	Gene	Gene Construct	Methodology	Variables	Disease	Long-Term Expression
Budker, V [8]	1998	Rat	Muscle	*b*-*Gal*, luciferase	Naked, Solution hypo/hypertonic	Artery injection High pressure (hind-limb)	Histochemistry, Luciferase	2 days	
Eastman, SJ [125]	2002	Rabbit	Liver	Alkaline Phosphatase Reporter gene	DNA	HD catheter lobar and whole liver	Plasma Alkaline Phosphatases	Model	2 days
Hagstrom, JE [93]	2004	Mouse, Rat, Dog, Primate	Muscle	DNA *Luc* vs. Ad; *EPO*; *Distr*	DNA, siRNA, Ad	HD vein limb	Luciferase	30 days	
Inoue, S [109]	2004	Rat	Liver	*b-Gal, luc-image, CTLA4Ig*	DNA dosing CTLA4Ig	HD system and local- catheter	Transplantation	2 days	
Zhang, X [113]	2004	Rat	Liver	Luciferase	DNA	HD vs. regional Portal	Luciferase	Method	short
Tosoulfas, G [117]	2006			*hAAT, Luc*	DNA	HD ex vivo DNA injection IVC closed	Injury, histopathology, physiology, efficacy	Transplantation	>5 days
Chang, H. [115]	2008			*IL10*-Ig fusion gene	DNA		IL-17, IL1beta, TNFa, IL1…	Myocarditis	
Suda, T [96]	2008	Mouse, Rat, Pig	Liver, Kidney, Muscle	*Luc, GFP, Ad-GFP*	DNA, Ad	HD computer assisted	Pressure, gene delivery/expression		
Xing, Y [119]	2009	Rat	Kidney	*Luc, EPO*		HD and ultrasound combination	Method		
Sawyer, GJ [120]	2010	Rat	Liver	*Luc*	DNA	HD-Regional Lobe without occlusion	Efficacy, luciferase activity	Method	
Wooddell, C [98]	2011	Mouse, Rat, Rhesus monkey	Muscle	*LacZ*	Plasmid DNA complexes	HD hind limb	Expression and delivery	null	49 weeks
Cim, A [121]	2012	Rat	Liver	*Pdx1, Ngn3, MafA*	5 different expression plasmids	HD	Expression, PCR, IHC	Diabetes type 1	28 days
Romero-Vasquez, F [138]	2012	Rat	Liver	hepatocyte growth factor	pCMV	HD	NFkB, RANTES, MCP1, IL6, oxidative stress	Renal hypertension	6 weeks with weekly treatment
Zhao, M [122]	2012	Rat	Endometrium	pigment epithelium derived factor	Polymeric micelle	intravenous injection	Clinic observation of endometrium lesions	Endometriosis	
Corridon, PR [139]	2013	Rat	Kidney	*eGFP, eGFP-actin/occluding/tubulin, tdTomato-H2B, RFP-actin*	Plasmid, adenovirus, baculovirus	HD retrograde renal vein	Expression-intravital, confocal	1 month	
De La Vega, J [140]	2013	Chinese hamster	Ovary cells	*GFP*	Plasmid lipofectamine lipoplexes	Methods of plasmid purification	Hydrodynamic diameter and zeta potential		
Yasuzaki, Y [124]	2013	Rat	Muscle	*Luc*	DNA	HD-hindlimb	Expression, luminescence, qPCR, WB	Method	24 h
Kamimura, K [126]	2014	Dog/Rat	Liver	*Luc, hAAT, hFIX*	Plasmid cDNA/DNA	HD-through hepatic veins of each 4 lobes with closed cava vein	Histology, physiological parameters	6 weeks	

**Table 3:** Hydrodynamic gene transfer works performed in large animals. The table shows the author of each work, the publication year, the animal model studied, the target organ assayed, gene and gene construction, injection methodology, variables evaluated, disease of interest and effect duration.

Author	Year	Species	Organ	Gene	Gene Construct	Methodology	Variables	Disease	Long-Term Expression
Hagstrom, JE [93]	2004	Mouse, Rat, Dog, Primate	Muscle	*DNA Luc vs. Ad; EPO; Distr*	DNA, siRNA, Ad	HD vein limb	Luciferase	30 days	
Yoshino, H [127]	2006	Pig	Liver	*GFP*, *CTLA4*-Ig	DNA	HD-cathe, closed (3 mg,150 mL, 5 mL/s)	Physiology, histology, fluorescence, plasma presence	Method	1 day (161 ng/mL)-7 days
Alino, SF [128]	2007	Pig	Liver (small vs. Large)	*hAAT*	DNA	HD-Cathe, open (100 mL, 7.5 mL/s)	ELISA, IHC, injury, qRT-PCR	hAAT deficiency	15 days (200 ng/mL)
Fabre, JW [129]	2008	Pig	Liver	*pGL3* plasmid, *Luc*	DNA	HD-isolated segment of IVC	Pressure, ECG, heart rate, luciferase activity	Method	1 day
Suda, T [96]	2008	Mouse, Rat, Pig	Liver, Kidney, Muscle	*Luc, GFP, Ad-GFP*	ADN, Ad	HD computer assisted	Pressure, gene delivery/expression		
Aliño, SF [18]	2010	Pig	Heart	*EGFP, GAPDH*	Naked	HD Cath Coronary sinus	IHC, PCR, RT-PCR, copy number	Method	1 day
Kamimura, K [130]	2010	Pig	Muscle	*pCMV-Luc*	DNA	HD hindlimb	Luciferase activity [95]	Method	60 days
Fabre, JW [131]	2011	Pig	Liver segment	*Luc*	DNA	Surg-HD-LivSeg portal vs. hepat vein	Vascular pressure (>100 mmHg)	Method	Short
Hackett, PB [84]	2011	Small&Large animals/Rev	Liver	*Luc*	Sleeping Beauty Transposon	HD	Integration, Plasma presence		
Wooddell, C [98]	2011	Mouse, Rat, Rhesus monkey	Muscle	*LacZ*	Plasmid DNA complexes	HD hind limb	Expression and delivery	49 weeks	
Carreño, O [132]	2013	Pig	Liver	*eGFP*	Plasmid cDNA	Surgery isolation, HD simultaneous	Expression PCR	1 day	
Zacharoulis, D [135]	2013	Pig	Liver	*eGFP*	Plasmid DNA vs. foamy virus vector-based	HD	Gene expression and qPCR	1 week to 1 month	
Sendra, L [133]	2014	Pig	Liver	*eGFP*	Plasmid cDNA	HD-surgical isolation cava vs. porta	Gene and protein expression, qPCR, ELISA, TEM	1 day	
Kamimura, K [141]	2015	Small and large animals	Liver	Various	Non-viral	HD		Various	
Sendra, L [19]	2016	Pig	Liver	*hAAT*	Plasmid DNA	HD-open vs. closed catheterism	Tissue expression qPCR, ELISA, clinic observations	hAAT deficiency	14 days

**Table 4:** Expression of hAAT-flag in human liver segments after hydrodynamic gene transfer. Preliminary results of hAAT-flag protein expression in human liver tissue after the hydrodynamic delivery of its gene. The first column of the table shows the number of human liver segment (HL1-4), second column indicates the total amount of hAAT protein in liver tissue (including hAAT and hAAT-flag) expressed in copy number per cell. The third column shows the specific amount of hAAT-flag protein. The fourth column represents the ratio between the amount of hAAT-flag protein respect to the total amount of hAAT as percentage.

Human Liver	Total hAAT (copy/cell)	hAAT-flag (copy/cell)	hAAT-f/Total hAAT (%)
1	7.16 × 10^5^	3.89 × 10^5^	54.31
2	8.98 × 10^5^	5.31 × 10^5^	59.18
3	9.62 × 10^5^	1.97 × 10^5^	20.46
4	4.03 × 10^5^	2.43 × 10^5^	60.13
Average	1.65 × 10^5^	8.86 × 10^5^	48.52
sd	1.59 × 10^5^	1.04 × 10^5^	18.88

**Table 5:** Hydrodynamic gene transfer works performed in human models. The table shows the author of each work, the publication year, the human model studied, the target cell or organ assayed, gene and gene construction, injection methodology, variables evaluated, disease of interest and effect duration.

Author	Year	Model	Organ/Cell	Gene	Gene Construct	Methodology	Variables	Disease	Long-Term Expression
Guillem, V [150]	2002	Human	Lymphoid cell line	*ODN*-FITC	CD3-PEI/ODN-	In vitro	Fluorescence, Cells increase	Method	
Guillem, V [151]	2002	Human	Jurkat & Granta	*eGFP*	CD3-PEI/eGFP	In vitro	Selective gene delivery	Method	
Lledo, S [152]	2005	Human	Cell line SW480	ASO-*Kras*	ASO phosphorotioates	In vitro	Cell viability	Cancer: colorectal	72 h
Lee, S [76]	2008	mouse/human cell	Liver	PTH	Plasmid DNA	HD	Plasma protein, expression	Hypoparathyroidism	
Diaz-Moscoso, A [153]	2011	Human, Mouse	Macrophage		80 nm manosilted cyclodextrin/DNAplex	In vitro	Delivery, FACS		
Doherty, J [85]	2012	Mouse/Human cell	Liver/HEK293, HeLa, T cells	Neomycin resistance cassette	transposone-piggybac	HD	Transgene expression		6 months
Herrero, MJ [145]	2012	Human	Liver	*eGFP*	pCMV	HD	Expression, PCR, fluorescence, IHC		2 days
Taniyama, Y [154]	2012	Human	Heart	Various	Plasmid	physical procedures		Various	
Balbino, TA [155]	2013	Human cells	HeLa		Cationic liposomes	Microfluidic systems comparison	Complex size, non-electrostatic bond, accessibility level		
Sevimli, S [156]	2013	Human	Cells HepG2, H460, SHEP, MRC5	*GFP*	Anionic and cationic polymers-siRNA	Transfection	Diameter, potential, stability, qPCR, WB, flow cytometry, confocal		
Matsui, H [36]	2014	Mouse/Human cell	Liver/HEK293	Full length Factor VIII	PiggyBac Transposon Vector cDNA	HD	Expression, PCR, qPCR, Coagulation assays	Haemophilia A	>300 days
Heller, R [157]	2015	Human	Various	Various	Non-viral	Electroporation	Clinical trials	Various	
Mendrek, B [158]	2015	Human	Cell line HT1080 (fibrosarcoma)		Plasmid-polyplex	Polyplexes DMAEMA (+) vs. DEGMA (0)	Hydrodynamic size, z potential, cytotoxicity, transfection efficacy		
